# Three decades of genetic privacy: a metaphoric journey

**DOI:** 10.1093/hmg/ddab164

**Published:** 2021-06-21

**Authors:** Bartha Maria Knoppers, Michael J S Beauvais

**Affiliations:** Centre of Genomics and Policy, Department of Human Genetics, Faculty of Medicine, McGill University, 740 av Dr Penfield, Montreal, Quebec H3A 0G1 Canada; Canada Research Chair in Law and Medicine; Centre of Genomics and Policy, Department of Human Genetics, Faculty of Medicine, McGill University, 740 av Dr Penfield, Montreal, Quebec H3A 0G1 Canada

## Abstract

Debates surrounding genetic privacy have taken on different forms over the past 30 years. Taking genetic privacy to mean an interest that individuals, families, or even communities have with respect to genetic information, we examine the metaphors used in these debates to chronicle the development of genetic privacy. In 1990–2000, we examine claims for ownership and of ‘humanity’ spurred by the launch of the Human Genome Project and related endeavors. In 2000–2010, we analyze the interface of law and ethics with research infrastructures such as biobanks, for which notions of citizenship and ‘public goods’ were central. In 2010–2020, we detail the relational turn of genetic privacy in response of large international research consortia and big data. Although each decade had its leading conceptions of genetic privacy, the subject is neither strictly chronological nor static. We conclude with reflections on the nature of genetic privacy and the necessity to bring together the unique and private genetic self with the human other.

## Introduction

In this retrospective, we have taken on the unenviable task of tracing the debates surrounding genetic privacy over the past 30 years or so. The task would surely be simpler if there were agreement about what this topic—privacy—is even about. Described as ‘exasperatingly vague and evanescent’, privacy frequently alludes precision, even with the best efforts (1). Its definition draws from the culture in which it emerges, with dignity and personhood founding many European conceptions and liberty—freedom from interference—founding many North American conceptions (2). More recently, articulating a conception of privacy from indigenous perspectives has also been the subject of increased interest (3,4). With privacy frequently rearing its head and posing challenges for the human genomics community, the implications of different conceptions have real-world consequences on the ability to successfully complete research projects and to translate scientific knowledge into the clinic.

We will not, and indeed cannot, detail the myriad nuances of the field, and are even less able to ‘solve’ the privacy issues genomics and its applications bring about. That is a Sisyphean task for another decade and for other authors. What we will do is present the leading privacy debates in genetic privacy over the past three decades. Our contention is that each decade’s debates had its own metaphors to assist in understanding genomics and then in regulating the activities where genetic privacy was implicated. Although both authors are former students of literature, the use of metaphor within law and ethics, across time periods and traditions, is so essential to the disciplines that it is difficult to conceive of law or ethics without it (5). No decade ever started with a clean slate; certain metaphors, certain conceptions were buried only to emerge again.

Before embarking, it is best to define what we understand genetic privacy to be. Genetic privacy is, at its core, an interest that individuals, families, or even communities have with respect to genetic information. These interests also ground claims with associated rights and obligations of ownership, of control, of exclusion, of non-interference, of protection, etc. We take an interest-based approach as it can encompass the breadth of the debates in the field over the past 30 years Genetic privacy may implicate an entire cast of actors—probands, their families, sequencing laboratories, researchers, clinicians, health system administrators, insurance companies and even public health agencies. The tricky issue for law and ethics is to determine which interests or claims should be recognized and reconciled among themselves. Central issues include: Do we give probands the sole prerogative to decide what happens with ‘their’ genetic information? Or do their families, or even communities, have a say? The metaphor used may give clues as to the intended outcome, a form of path dependency.

## Ownership and ‘Humanity’ (1990–2000)

The launch of the Human Genome Project in 1990 was a catalyst for increased attention and thinking about the ethical and legal consequences of genomics. It was clear that genetic data would play an increased role in the biosciences and within society more broadly. One scholar, for example, posed a portentous question—will genetic information ‘impel us to take privacy much more seriously in the genetic realm … or lead us to give up on maintaining personal privacy altogether’ (6)? In a way, the privacy concerns regarding genetic data presaged our contemporary debates about the intersection of privacy, surveillance and infrastructures. It was not only that genetic data were novel in the abstract. They also gave rise to new forms of infrastructures such as sequence maps, genetic databases and then biobanks.

There was early recognition that genetic data were different from other kinds of medical data (6–8). The data are probabilistic, they have implications for blood relatives, they escape our ‘full’ understanding (and continue to do so), etc. Indeed, a stumbling block during this time was getting the science right—many commentators suffered from a poor understanding of genetics. A common misstep was to overestimate, with seemingly unbridled enthusiasm (or profound dread) the ability of genetic data to unlock the presumed totality of our biological and social existence (9). Through this lens of genetic exceptionalism, the secrecy and seclusion of data were emphasized. Even secrecy from oneself, in the form of the ‘right not to know’, was recognized (10,11). Often there was a dichotomy between large-scale sequencing and privacy, which commentators used to argue that it was impossible to maintain privacy when so much data would be generated about individuals. (8).

More modestly, were genetic data ‘an individual’s probabilistic future diary’ (6)? Or perhaps they were one’s property? The State of Florida thought so—the legislature decided that genetic data were ‘the exclusive property of the person tested.’ (12) The property approach stresses the right to exclude others from and control over an object, allowing for its sale and exchange. From this perspective, the proband could claim control over the uses of their genetic data, and even have a commercial interest in their tissues and data (13). The decisional power in this regard was almost universally located in the proband, with some tailored exceptions, e.g. for determinations as to paternity (7,14). The genetic property approach managed to somewhat flourish despite recognition in both common law and civil law jurisdictions that parts of the human body were not property (15,16).

The property approach was just one of an array of approaches. By 1995, and before the enactment of the USA’s *Health Insurance Portability and Accountability Act of 1996*, at least eight states had legally recognized a genetic privacy interest (8). Fifteen had enacted laws curtailing the use of genetic data for insurance purposes (17). Comparatively, less specificity was afforded to genetic data in Europe. This was not because privacy was not taken seriously. To the contrary, a comprehensive privacy law that applied to personal data held by public and private entities alike, the *Data Protection Directive*, the predecessor of the *General Data Protection Regulation*, included genetic data in its ambit through regulating personal health data (18). Although, other normative instruments in Europe also aimed to ensure that genetic data were only collected and used for bonafide clinical and research purposes (10,19).

Irrespective, the human genome at the species level received another legal qualification. As early as 1991, it was argued that the human genome *per se* was beyond the control of any single individual, corporation or government—it was the common heritage of humanity (20). There was no right to exclude others from knowledge of the human genome. In this vein, the 1996 Bermuda Principles did much to foster a culture of sharing and openness of data, which likely would have otherwise crumbled under the weight of the contemporaneous patent wars over human genes (21,22). The year 1997 saw the issuance of UNESCO’s *Universal Declaration on the Human Genome and Human Rights*, which declared the human genome to symbolically be the heritage of humanity (11).

## Citizenship and ‘Public Goods’ (2000–2010)

The announcement of the completion of the first draft human genome sequence from the International Human Genome Sequencing Consortium in 2000 was a feat of international and public–private collaboration. Followed by the International HapMap Consortium (23) and the 1000 Genomes Project Consortium (24), large international consortia became the norm for much genomic research, but not without some growing pains regarding both regulatory and scientific harmonization. During this decade, we begin to see more clearly the dance of law and ethics with research infrastructure. Central for genetic privacy were discourses that linked one’s genomic data to scientific participation in the building of infrastructures for discovery science, thereby infusing it with notions of scientific citizenship (25).

Population biobanks, which store biosamples and data of large numbers of individuals for future, unspecified research, were established in Estonia (26), Canada (27,28), Japan (29), the UK (30) and elsewhere. These large repositories were conceived of as longitudinal resources for future research and managed in the common interest. These projects posed complications for genetic privacy and the consent-laden property approach. To continue with the property metaphor, individual consent makes sense for managing one’s own objects or patrimony, as it is known in the civil law tradition. But global public goods, as human genomic databases were intended to be (31), require governance beyond the level of the individual.

Yet, it became apparent that unanchoring genetics from the individual would not be possible. In 2003, the Supreme Court of Iceland recognized a claimant’s privacy interest in her deceased father’s medical data, challenging the presumed consent introduced by the country’s *Health Sector Database Act*, and presaging the relational accounts of privacy to come (32). Despite the creative legal fiction of presumed consent *en masse*, some form of meaningful consent remained indispensable for research infrastructures (33).

With the groundbreaking finding that individuals could be reidentified in summary statistics with just a modest number of SNPs, the place of the individual arguably became even more important in this decade (34). Even so, the justificatory force of individual consent and autonomy began to show their limits when it came to genetic data. Some argued that the complex implications of genetic data meant that orthodox approaches to consent were not up to the task of ethically justifying genetic research on diseases, to say nothing of biobanks (35). It was further recognized that property-based discourses ‘negate other social meanings of the body [and of genetics]’, such as its communal and familial significance (36).

Reconciling an ethos of the public good with individual autonomy and privacy was not easy. Trenchant debates, in particular around broad consent, (37–39) left an indelible mark on genetic privacy that still influences us today. These debates were able to evade an epistemic knot through explicitly focusing on the governance of the infrastructures in which genetic privacy interests were located. Biobanking guidelines emphasized transparency, integrity of purpose, justification for the use of personal data, ongoing ethics governance, prohibitions on discrimination and other such safeguards (40). These mechanisms provided an alternative way of attending to the presumed dangers of broad consent to future research.

An account of genetic privacy during this decade cannot ignore the sweeping changes that ‘Web 2.0’ brought about, which itself stressed individual participation in communities supported by technical infrastructures (41). Social media brought new communities together, notably those with rare diseases, who often found that more knowledge was held among members of an online group dedicated to their malady than within their circle of clinicians (42). Cloud computing further enabled more sophisticated applications—from smartphone applications to bioinformatics pipelines—but buried the seeds for seemingly intractable geographical and jurisdictional issues to come (43). If genetic data were an aspect of scientific citizenship, they would still require a political ‘home’.

## Relationships and ‘Genetic Data’ (2010–2020)

Faced with scandals such as the Snowden revelations (44,45) and Cambridge Analytica (46), the public’s concerns about privacy have increased (47,48). Developments such as the *Schrems I* (49) and *Schrems II* (50) decisions—direct consequences of the Snowden revelations—have rendered data sharing outside of Europe markedly more difficult (51,52). Yet, the complexity of the science, ethics and law has done little to stifle ambitions. In this decade, the Global Alliance for Genomics and Health (GA4GH) (53), International Rare Diseases Research Consortium (54) and the International Cancer Genome Consortium’s Accelerating Research in Genomic Oncology (55) were launched, with more participants and jurisdictions involved than what seemed possible in the past. In the face of such developments, genetic privacy has taken a relational turn.

In keeping with the complex changes brought about by network societies, direct-to-consumer (DTC) genetic testing brought about its own major shift in context—genetics was taken from the lab to the home. Genetic data became yet another medium through which relationships were expressed. Individuals’ genetic privacy fell into their own hands and challenged the largely paternalistic lens of biomedical ethics (56). With DTC sequencing and interpretation services, it has been noted that new kinds of regulation are needed to ensure adequate privacy protection but also to ensure safety and quality of such services (57).

Continued reliance on consent suggests that proprietary understandings of genetic privacy, in some form, are here to stay (58). Despite our own practical and legal objections to the notion of genetic property, the language and narrative of ownership in genetic and health data resonates with many. What ‘ownership’ connotes shifts in response to context and speaker, and attention is warranted (59). Even the generally exclusionary nature of ownership can be understood in terms that emphasizes the qualities of relationships we want to promote (60).

After some decades of ‘genetic privacy’ concerns, some have dismissed its distinctiveness entirely, instead underscoring that the issues genetic data pose are little different from ethical and legal inquiries regarding rights and obligations and of living together (61). Others have relatedly labeled meaningful individual control of genetic as ‘a mirage’ that distracts from the core questions of which individual and social goods should be realized (62). Even those who opine that genetic privacy remains a valuable, distinct concept have called for the need for ‘epistemic modesty’ to recognize that genes and other biomarkers only give a limited picture of a person, telling us nothing about the ways in which individuals self-identify (63).

The advent of big data has seen individuals constructed into groups of which they are unaware, much less self-identify (64). Consider polygenic risk scores. With an increasingly strong evidence base, they are enabling new ways of stratifying the population into tiers of risk for certain diseases, e.g. breast cancer (65). Individuals may be wholly unaware of their risk group but nevertheless affected by the use of another individual’s data in the same subpopulation (66). Ensuring the proper use of data with group implications challenges the individualistic approach of privacy law. A shift in thinking that focuses on obligations instead of rights is a start (66). As the openness of data is increasingly emphasized, more debate is required on the proper balance between individuals, groups and society and on the best way to maintain public trust.

Obligations may indeed be better suited to issues such as familial interests in genetic information—the proband has independent privacy rights but also, in certain circumstances, the responsibility to consult with relevant family members. Although ethics has little issue recognizing such obligations (67), the law has proven slow to give up on its image of the fully autonomous person. Nevertheless, relational approaches to genetic privacy have found explicit recognition in England and Wales. There, the High Court recognized that clinicians have a legal duty to consider the interests of their patient’s genetic relatives in deciding whether to disclose health information that could reduce or prevent a serious risk of harm from materializing (68). More simply, the individual is not the only person with an interest in ‘their’ genetic data, a proposition that science and ethics had recognized decades ago. Time will tell whether other jurisdictions follow the relational lead.

## Conclusion

Our study of genetic privacy spanning the last 30 years reveals approaches that are neither strictly chronological nor static. With more facets of social life rooted in information flows, privacy, genetic or otherwise, finds itself interacting with other incommensurables. Indeed, the progressive realization of human rights, such as the human right to science, hinge upon the sharing of data (69). Societies and individuals have choices to make. The recent advent of whole genome sequencing has further placed the individual into a context of choice—what to share, what to control and what to contribute to research and international, collaborative efforts. What has changed however and will continue to do so is the image of genetics as limited to rare diseases and running in fated families and with a strictly personal and private character.

Today, when digitized and presented as subpopulations, as polygenic risk scores in common diseases, as predisposition, susceptibility or resistance to risk, or, as epigenetic lifestyle risks, the question becomes less one of individual genetic privacy than one of groups and relationships that demonstrate genomic equivalence in difference. As genetic testing becomes commonplace, formerly ‘private’ genetic information will be shared as any other type of health information in the clinical care of patients and their families. The success of precision medicine hinges on facilitating contributions to the healthcare system to promote the validation and interpretation of the health implications of individual variants. Such a systemic approach to ownership, citizenship and relationships must promote the right of everyone to benefit. In short, the future genetic self is necessarily linked to others. The unique and private genetic ‘me’ is discovered through the study and comprehension of the human ‘us’. *Conflict of Interest statement*. None declared.
